# Dieulafoy disease with gastric MALT lymphoma: A case report

**DOI:** 10.1097/MD.0000000000022651

**Published:** 2020-10-09

**Authors:** Qin Zeng, Jin Feng Dai, Haijun Cao, Shuo Zhang

**Affiliations:** a Zhejiang University of Traditional Chinese Medicine; b Department of Gastroenterology, the First Affiliated Hospital of Zhejiang Chinese Medical University, Shangcheng District, Hangzhou, Zhejiang Province, China.

**Keywords:** Dieulafoy lesion, endoscopic hemostasis, mucosa-associated lymphoid tissue lymphoma, over-the-scope clip, upper gastrointestinal hemorrhage ;

## Abstract

**Rationale:**

Dieulafoy lesion (DL), a rare cause of gastrointestinal bleeding, is easily covered by blood scab formation on the mucous membrane for its small size, which makes it difficult to be identified under endoscope. In clinical practice, it is also very easy to miss gastric mucosa-associated lymphoid tissue (MALT) lymphoma that exhibits atypical early manifestations under gastroendoscope and is difficult to be diagnosed by routine superficial biopsy. Most patients only experience nonspecific dyspepsia symptoms.

**Patient concerns:**

A 68-year-old man suffering from repeated melena for 6 years arrived at our hospital. The patient had undergone gastroscopy and capsule endoscopy at other hospitals for several times and received symptomatic treatment, but his melena still continued to recur. At our hospital, the capsule endoscopy displayed that there existed large hemorrhage in the stomach, after which a gastrointestinal decompression tube was placed, so the bright red blood was drained. Subsequently, a sunken vascular malformation tissue in the anterior wall of the gastric fundus was observed under emergency endoscope. Pulsating blood flow appeared immediately after biopsy, and over-the-scope clip (OTSC) was quickly applied to stop the bleeding. Near the bleeding point, scar-like tissue that was surrounded by interrupted mucosa was discovered, and biopsy was performed at this site.

**Diagnosis:**

The diagnosis of DL and gastric MALT were determined by the digestive endoscopy and biopsy pathology.

**Interventions:**

With the diagnosis of DL and gastric MALT, the hemorrhagic spot was treated by OTSC. After the patient's condition was stable, anti-*Helicobacter pylori* treatment was performed.

**Outcomes:**

After the corresponding treatment, the 6-month follow-up revealed that the lymphoma was not completely cured, but no further bleeding occurred. There was no bleeding in the epigastric region and the patient was in good condition.

**Lessons:**

From endoscopy, it is easy to miss DL. When the hemostatic equipment is fully prepared, biopsy can be performed. After biopsy, pulsatile bleeding is convincing evidence for Dieulafoy disease. OTSC represents an effective and low-risk method for DL and it could replace surgery. Moreover, the mucosa surrounding Dieulafoy disease should be carefully observed to exclude coexisting diseases such as lymphoma or gastric cancer.

## 1 Introduction

Dieulafoy lesion (DL) is a rare cause of gastrointestinal hemorrhage, accounting for approximately 1% to 2% of acute gastrointestinal bleeding causes.^[1]^ Among them, the proportion of elderly patients is higher, and more men are affected than women.^[2]^ Approximately 60% of the bleeding lesions are located 6 cm away from the junction of the esophagus and heart on the side of the lesser curvature in the stomach, around 12% in the antrum of the stomach. The remaining cases are likely to be in the ileum or colon.^[3]^ Due to the rapid arterial retraction and mucosal repair after Dieulafoy disease-related hemorrhage, hemorrhage lesions may not be found even after the endoscopic examination. Therefore, the clinical diagnosis rate is low, and multiple endoscopic examinations are required for diagnosis. According to Reilly HR et al,^[4]^ among 66 cases suspected DL diagnosis, only 49% was detected at the first endoscopy, 33% required multiple endoscopies, and some required surgery for the confirmation. The disease can cause anaemia and is life-threatening in severe cases, and even lead to hypovolemic shock, with a clinical mortality rate of up to 80%.^[5]^ This article reported a case of repeated upper gastrointestinal bleeding and a final diagnosis of DL in a gastric MALT lymphoma-treated patient.

## 2 Case presentation

**Figure 1:**
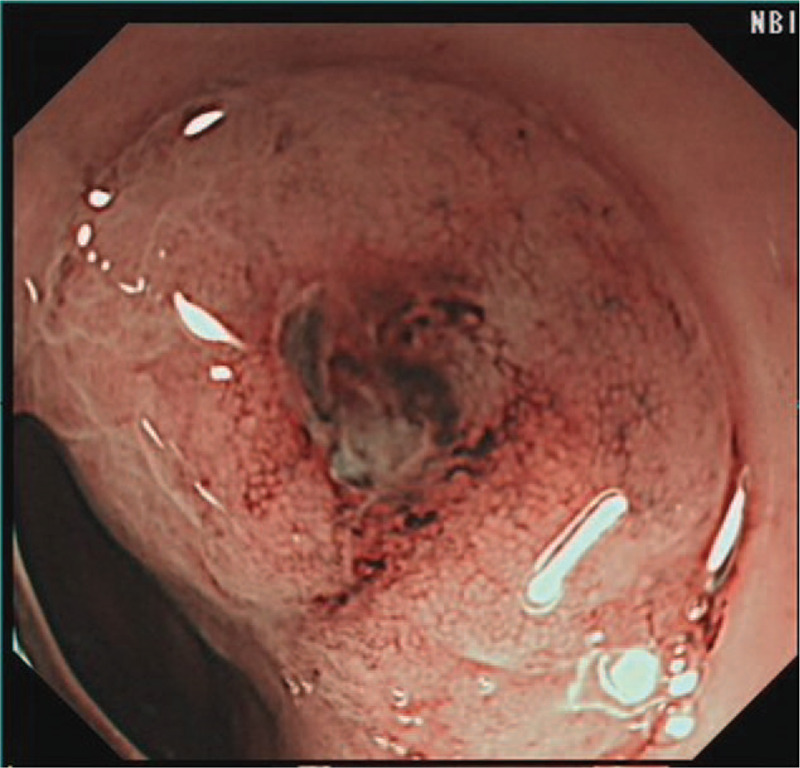
An abnormal blood vessel with a diameter of approximately 0.8 cm in the anterior wall of the gastric fundus.

**Figure 2:**
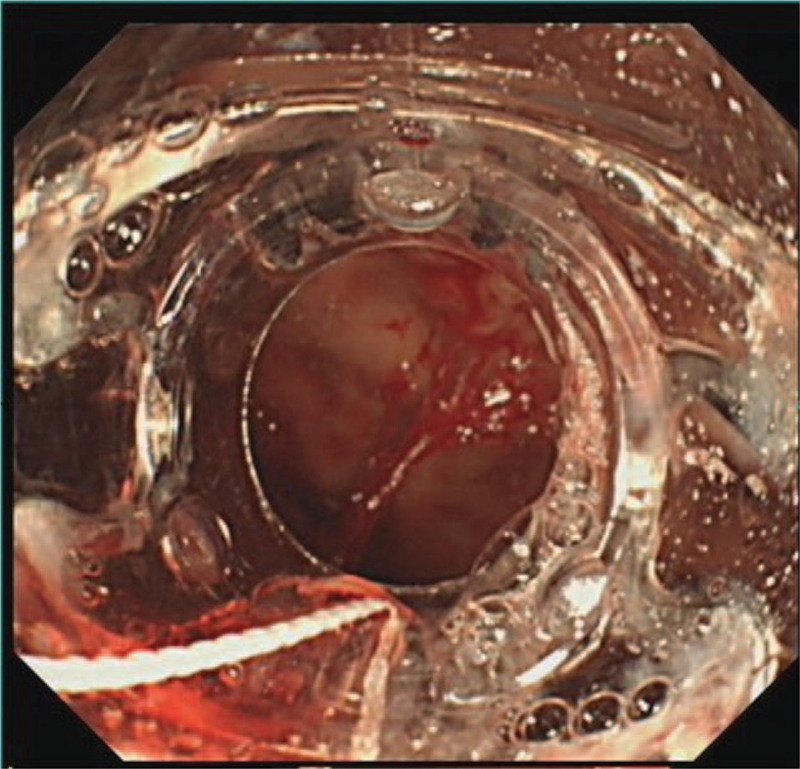
Pulsating blood flow was observed after biopsy using forceps, and an over-the-scope clip was used to clip the bleeding tissue.

**Figure 3:**
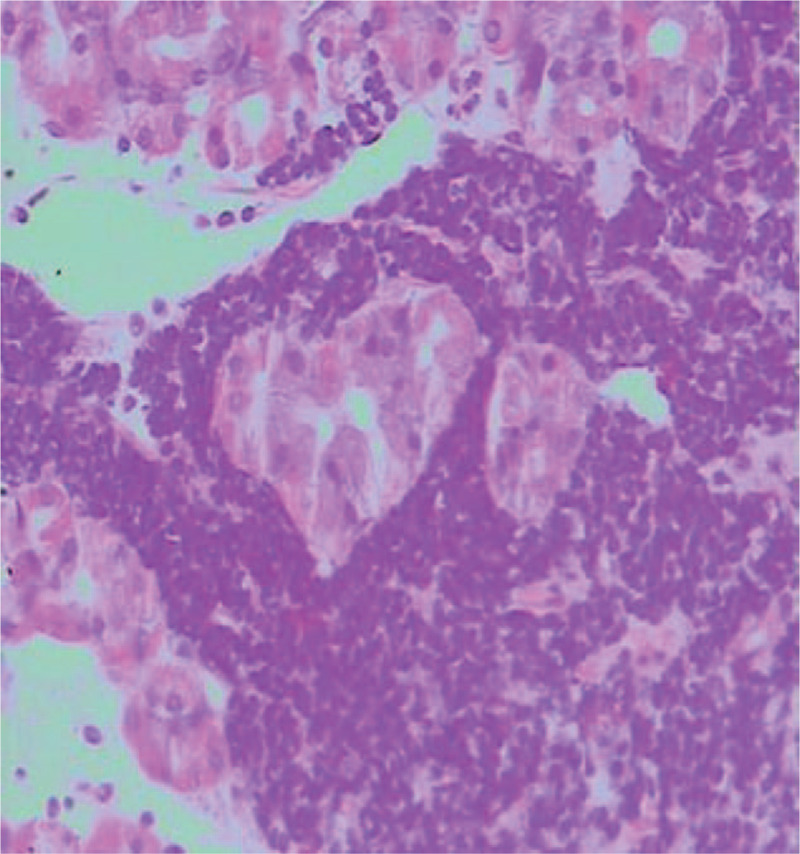
Chronic inflammation of subcardia mucosa was observed, along with moderate chronic superficial gastritis of the gastric fundus (active) accompanied by abnormal hyperplasia and erosion of lymphoid tissues.

**Figure 4:**
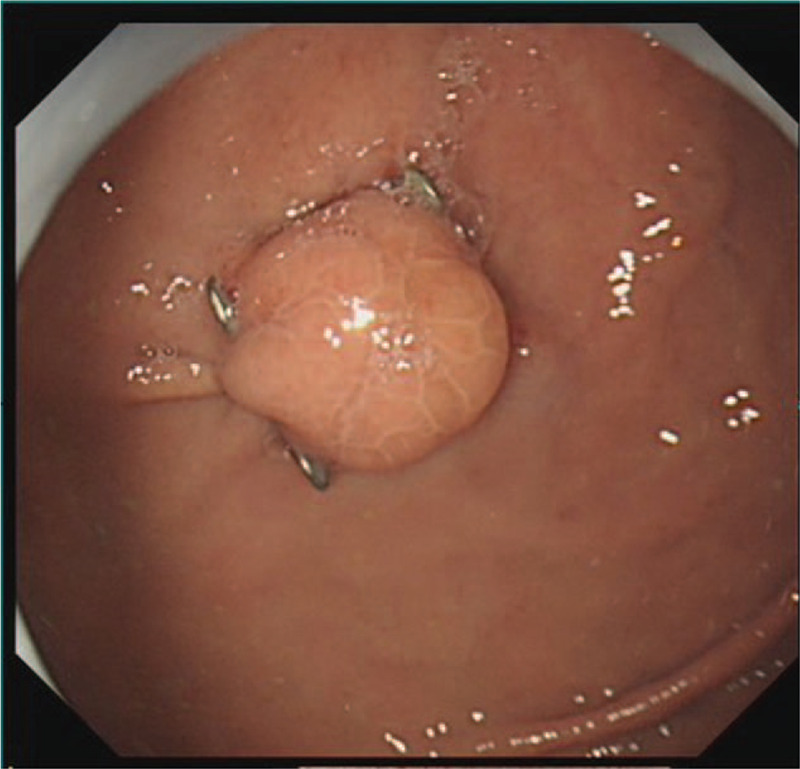
The over-the-scope clip could be seen in the greater curvature of the gastric fundus, the mucosa in the anterior wall was slightly concave and white, and the peripheral plica was slightly uplifted.

**Figure 5:**
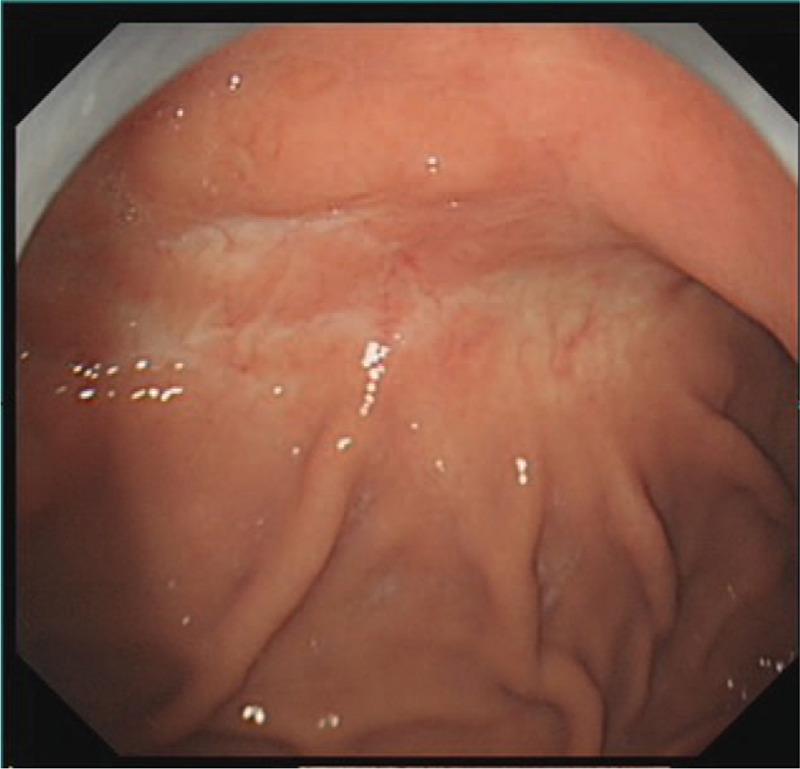
The mucosa was slightly dented in the anterior wall of the stomach, with concentrated peripheral folds and a slightly congested surface. Biopsies were taken at the anterior wall and the anterior wall near the gastric fundus.

**Figure 6:**
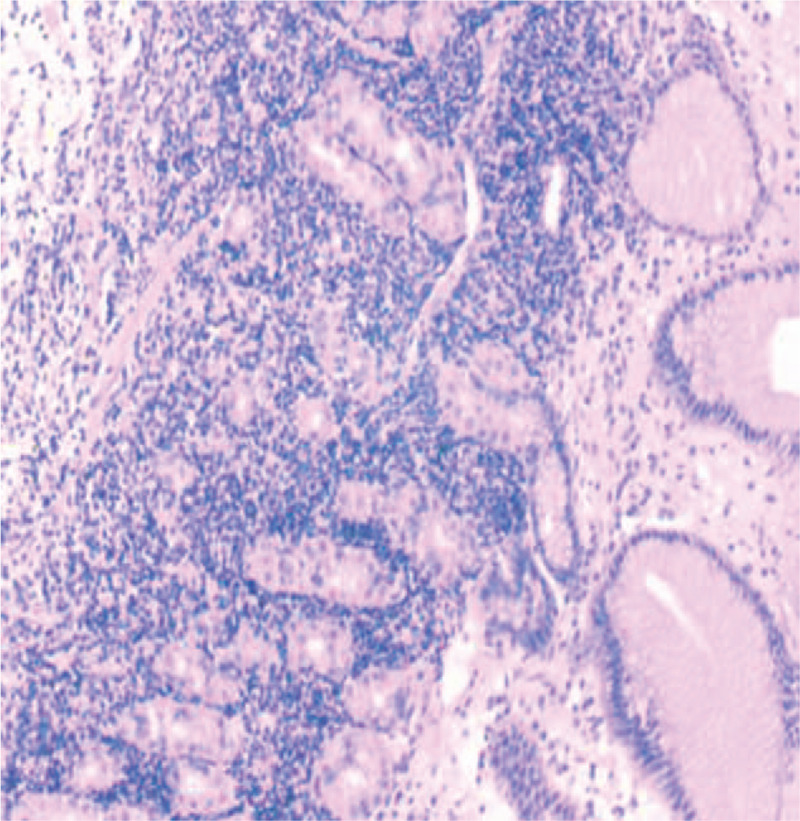
Dysplasia of mucosal endolymphatic tissue in the anterior wall of the stomach near the gastric fundus, and B-cell lymphoma of mucosa-associated lymphoid tissue was considered based on these results combined with HE staining and immunohistochemistry, which showed mild chronic superficial gastritis with interstitial oedema in the anterior wall of the stomach and gastric fundus mild chronic superficial gastritis with interstitial oedema; HP (−).

A 68-year-old man suffered from black stool for 6 years. He was treated with acid inhibition and erythrocyte transfusion once at other hospitals, because no bleeding lesions were found during gastroscopy or capsule endoscopy. Physical examination revealed a pale complexion and stable vital signs, indicating moderate anaemia, and normal medical examination results. The laboratory examination results illustrated the blood analysis of a white blood cell count of 2.7 × 109/L, a red blood cell count of 2.81 × 1012/L, hemoglobin level of 78 g/L, and urea nitrogen level of 8.39 mmol/L. No obvious abnormality was found in other laboratory examination results. The gastroscopy results displayed no significant abnormality, and no bleeding lesion was observed. In order to confirm the cause of bleeding, capsule endoscopy was carried out on the same day, and the position of the endoscopic capsule could be evaluated within 24 hours after the patient swallowed it. A large amount of fresh blood was observed in the stomach, and the patient was recommended for the immediate gastroscopy. The patient exhibited anhidrosis in the early morning, and then a gastrointestinal decompression tube was placed, so the bright red liquid was drained. The NBI + ME results demonstrated an abnormal blood vessel in the anterior wall of the gastric fundus (Fig. 1). Pulsating blood flow was observed after biopsy, and an over-the-scope clip (OTSC) was applied to clip the bleeding tissue (Fig. 2). Abnormal small lymphocyte diffuse hyperplasia was discovered in the mucosa of the anterior wall of the stomach. The combination immunohistochemistry results could not exclude mucosa-associated lymphoid tissue (MALT) lymphoma, and the combination immunohistochemistry results of the gastric fundus could not exclude MALT lymphoma (Fig. 3). After the patient's condition was stable, the anti*-Helicobacter pylori* treatment, including enteric-coated pantoprazole capsule Po 40 mg bid, colloid pectinate bismuth capsule Po 200 mg bid, clarithromycin tablet Po 500 mg bid, and amoxicillin capsule Po 1000 mg bid for 2 weeks were conducted. Blood analysis proved a white blood cell count of 2.9 × 109/L, a red blood cell count of 2.89 × 1012/L, and a hemoglobin level of 81 g/L. After 3 months, the OTSC could be observed in the greater curvature of the gastric fundus (Fig. 4). The mucosa was slightly dented in the anterior wall of the stomach, with concentrated peripheral folds and a slightly congested surface (Fig. 5). The pathological findings, combined with HE staining and immunohistochemistry, gave information that the B-cell lymphoma of MALT was HP(-) (Fig. 6). Thus far, no gastrointestinal bleeding has been observed in the patient. Blood analysis proved a white blood cell count of 3.6 × 109/L, red blood cell count of 4.2 × 1012/L, and hemoglobin level of 113 g/L. Observation is recommended for the patient. The patient was followed up after half a year and 1 year respectively. There was no bleeding in the epigastric region and the patient was in good condition.

## 3 Discussion

DL was first reported by Gallard in 1884 and then described in details by Georges Dieulafoy in 1898. Although DL is a rare cause of gastrointestinal bleeding, it is extremely destructive. The amount of blood loss is large, because the arterial bleeding rate occurs at a rapid rate, which can easily lead to hypovolemic shock, and can even be life-threatening if not treated in time. The clinical manifestations are generally black stool, hematemesis, and hypotension. DL is difficult to identify and diagnose via endoscopy, because the small lesions in the superficial mucosa are often covered by blood clots, thus resulting in a low clinical diagnosis rate.^[2,6]^ Previous studies reported the interaction between Dieulafoy and gastrointestinal cancer. As early as 1992, DeVault Kr et al^[7]^ reported a case of acute Dieulafoy hemorrhage in distal esophagus that could not be stopped by endoscopy. After thoracotomy, schwannoma was found at the bleeding site, and it was related to the failure of hemostasis for the repeated bleeding. In addition, the literature points out that the occurrence of gastric tumour is closely related to the repeated bleeding. By reporting a case of Dieulafoy disease and early gastric cancer, Leone O et al^[8]^ proposed that the gastric wall abnormality is the result of vascular malformation that may destroy the nutrition of gastric wall. In addition, in the context of mucosal regeneration in ischemic injury related to vascular malformation, “signet ring cell dripping of early gastric cancer should be considered. According to a case and literature review of Dieulafoy with type IIa gastric cancer, taketsuka S et al^[9]^ pointed out that the dyscirculation in Dieulafoy blood vessels can cause repeated mucosal regeneration. The recurrent regeneration of vascular mucosa and abnormal hyperplasia of mucosa may be 2 of the factors to promote gastric cancer. Similarly, gastric lymphoma is often overlooked under gastroscope. Gastric malignant lymphoma accounts for 5% to 10% of all gastric malignant tumours, while gastric MALT lymphoma occupies 50% of gastric malignant lymphoma. The age of disease onset is 50 to 60 years old, with no different prevalence between men and women.^[10–12]^ More than 90% of gastric MALT lymphomas are associated with *H. pylori* infection.^[10]^ Clinical symptoms of gastric MALT lymphoma are not typical. Patients only present nonspecific dyspepsia symptoms, including nausea, vomit, and abdominal distension, and endoscopic lesions are also not typical. MALT lymphoma originates from B cells in the posterior margin of the lymphoblastic follicular germinal centre. Based on superficial biopsy pathology, the diagnosis is difficult, with low clinical diagnosis rate. The diagnosis also depends on other symptoms and auxiliary examinations. Related reports indicated that it is frequently diagnosed as an advanced tumour, and misdiagnosis and excessive treatment can occur.^[13]^ Common endoscopic manifestations of gastric MALT lymphoma include ulcers (45%), intragastric nodules (30%) and pseudogastritis (25%).^[14]^ The endoscopic findings of a diffuse miliary appearance of gastric mucosa, with slightly white and small protrusions, were reported in 2 cases, and nodular gastric MALT lymphoma was considered and pathologically confirmed.^[15]^ Endoscopic ultrasonography is helpful for the diagnosis and treatment of lymphoma, because the invasion depth of lymphoma is also closely related to the development of the disease,.^[16]^ The elimination of *H. pylori* can effectively cure early gastric MALT lymphoma.^[17]^ In terms of both gastric MALT lymphoma and DL, the clinical symptoms are not significant and easy to be missed. The former is a malignant tumour. If it is not detected in time, it could enter into an advanced stage, carrying a poor prognosis. The latter is also urgent and quite dangerous. An untimely diagnosis increases the mortality rate of this disease. In the case reported herein, both DL and MALT gastric lymphoma were explained. In terms of the patient who reported repeated black stools for 6 years, no bleeding lesions were detected by gastroscopy at other hospitals, and no lymphoma lesions were found. This case illustrated that physicians who treat patients with recurrent gastrointestinal bleeding upon endoscopic examination should be highly alert to Dieulafoy disease and other coexisting diseases.

Unfortunately, currently, there exist no definite guidelines of the clinical treatment for DL, and endoscopic treatment is considered as an effective treatment scheme for DL.^[18]^ The main hemostasis methods of endoscopic coagulation include injection drugs (such as adrenalin and sclerotherapy), ablation (coagulation via heat, electrocoagulation, argon plasma coagulation, etc), vascular clamp, and mechanical ligation.^[19]^ Some scholars utilize endoscopic injection therapy or endoscopic band ligation (EBL) for the treatment of DL that can successfully stop bleeding, but the endoscopic injection therapy rebleeding rate is higher than that of EBL.^[20]^ A meta-analysis assessed the rate of DL treatment using EBL versus an endoscopic hemo clip, suggesting no significant differences between these 2 approaches.^[21]^ The effective hemostasis rate of epinephrine, sclerotherapy, and ablation were found to be lower than that of mechanical hemostasis. The method of OSTC application under endoscope is a kind of mechanical ligation method, and OSTC is a multitoothed clip, composing of a nitinol alloy. OSTC can close the damaged mucosa throughout the whole layer and has great grasping power, which has new significance for the emergent treatment of nonvaricose gastrointestinal bleeding. OTSC has caught the attention of endoscopists, since its introduction in 2007. Matthew Skinner et al adopted an OTSC for hemostasis in 12 patients suffering from recurrent acute gastrointestinal bleeding. On the first and seventh days after OTSC placement, only 2 patients experienced rebleeding, while the rest did not. None of the treated patients had obvious complications.^[22]^ Ravishankar Asokkumar et al reported that the success rate of OTSC in endoscopic hemostasis of high-risk nonvaricose upper gastrointestinal bleeding was 100%, including in 4 cases of DL bleeding and 18 cases of 19 nonvaricose bleeding lesions via endoscopic treatment.^[23]^ A meta-analysis followed up 1865 patients. The results revealed that OTSC had an overall technical success rate of 93.0% for gastrointestinal bleeding and a long-term clinical follow-up success rate of 87.5%, which confirmed that OTSC was effective for hemostatic closure of gastrointestinal bleeding.^[24]^ In the present case, the patient was repeatedly treated with endoscopic tissue clips and drugs at other hospitals, but the effects of the treatments were not obvious. OTSC was applied to stop the bleeding, and no rebleeding recurred for 6 months. Hence, the effect of this treatment was remarkably better than that of ordinary tissue clips. Therefore, we believe that OTSC can be adopted as an effective treatment for DL and can replace surgery to some extent.

DLs are easily overlooked under endoscope, and biopsy can be performed when the hemostatic equipment is fully prepared. Pulsatile bleeding after biopsy is indicative of DLs. OTSC application marks an effective and low-risk method to treat DLs and it has the potential to replace surgery. Most importantly, the mucosa surrounding Dieulafoy disease should be carefully observed to avoid overlooking other coexisting diseases, such as lymphoma or gastric cancer.

## Acknowledgment

We wish to thank the colleagues in the pathology laboratories and endoscopy centre at the First Affiliated Hospital of Zhejiang Chinese Medical University for their excellent technical assistance. Special thanks to Dr Zhang CL for their expert pathologic opinions. We thank the patient and his family for their kind cooperation.

## Author contributions

Zhang Shuo and Cao Haijun were responsible for surgery and finding the lesions. Zeng Qin and Dai Jin Feng were responsible for consulting the relevant literature on malt lymphoma and Dieulafoy lesions.

**Conceptualization:** Shuo Zhang, Haijun Cao, Qin Zeng and Jinfeng Dai.

**Data curation:** Jin Feng Dai, Haijun Cao.

**Formal analysis:** Haijun Cao.

**Resources:** Shuo Zhang.

**Supervision:** Shuo Zhang.

**Writing – original draft:** Qin Zeng, Jin Feng Dai, Shuo Zhang.

**Writing – review & editing:** Qin Zeng.

## Abbreviations

Abbreviations: DL = Dieulafoy lesion, EBL = endoscopic band ligation, MALT = mucosa-associated lymphoid tissue, OTSC = over-the-scope clip.
